# Health sciences faculty publication patterns and related information-seeking behavior

**DOI:** 10.5195/jmla.2024.1789

**Published:** 2024-04-01

**Authors:** Sandy De Groote, Jung Mi Scoulas

**Affiliations:** 1 sgroote@uic.edu, Professor and Head, Assessment & Scholarly Communications, University Library, University of Illinois Chicago, Chicago, IL; 2 jscoul2@uic.edu, Assistant Professor and Assessment Coordinator, University Library, University of Illinois Chicago, Chicago, IL

**Keywords:** Faculty, Research Practices, Academic Libraries, Faculty publications

## Abstract

**Objectives:**

This study aims to explore how health science faculty publication patterns at a large public research university have changed over time and examine how productivity relates to their information-seeking behavior and perception of the academic library.

**Methods:**

Two datasets were utilized: one consisted of publication records of health sciences faculty spanning a 15-year period, while the other was from a faculty survey exploring faculty's perception of and satisfaction with library resources and services related to their research.

**Results:**

Health sciences faculty publication patterns have changed over time, characterized by greater productivity, collaboration, and use of literature in their publications. Faculty's literature use correlates with productivity, as evidenced by both datasets. The survey revealed that faculty with more publications tend to rely more on online journals and Interlibrary Loan (ILL). Similarly, the publication data indicated that less productive faculty tended to use fewer references in their publications.

**Discussion:**

The publication data and survey results offer valuable insights into the health sciences faculty's information-seeking behavior and productivity. Online access to information has been effective in facilitating use of information, as indicated by the greater incorporation of references in publications.

**Conclusion:**

The study highlights the changing publication patterns and productivity of health sciences faculty, as well as the role academic libraries play in supporting their research and publishing activities. Although multiple variables influence faculty access to and use of information, faculty attitudes towards the library and use of the library are related to faculty research and productivity.

## INTRODUCTION

The academic health sciences library and the information-seeking behaviors of its users have changed significantly as new online resources and formats have become available. Over two decades of research has shown how online resource usage has overtaken print resources in use and preference [1–4]. Many researchers have also shifted from using Abstracting and Indexing (A&I) databases such as MEDLINE as the main resource for locating literature toward using Google and Google Scholar, at least as the initial starting point [5–8]. Health Sciences faculty have reported how the growth of online information has changed how they searched for and accessed articles, allowing them to expand interests, read more peripheral articles, read articles more in-depth, read a greater number of articles, expand the disciplinary range of journals used, and improve their ability to stay current [7].

The impact of online information is evident within citation patterns in the published literature. During the transition period from print to online journals, references from journal articles that were only available in print had dropped in certain disciplines (nursing and dentistry), while the use of journal articles available in an online format increased [9]. A 2008 study also observed that as more journals and databases became available online, the number of references included in journal articles written by medical college faculty also increased [10]. While online journals and databases are now ubiquitous, health sciences faculty continue to rely on library resources for student lecture preparation and conducting research and highly valued online journals, databases, electronic books, inter-library loan and library personnel in support of their research [11]. Studies have also demonstrated a relationship between the reading of scholarly articles and faculty engagement in research and productivity [2]. While previous studies demonstrate faculty's reliance on academic libraries and how their use of library shaped on their research productivity, it becomes imperative to take investigations a step further and explore if the perceived value and use of library resources and/or the use of scholarly articles in publications correlates with faculty productivity.

This paper reports on the findings of a survey distributed to health sciences faculty to determine how their use of and perceptions of academic library resources and services relates to their research. In addition, using health sciences faculty journal article publication data, it also explores how publication patterns have changed over time and the relationship between health sciences faculty productivity and their use of literature in journal articles.

## METHODS

Data from two different data collection projects are presented in this paper. This includes a retrospective collection of publication data from journal articles authored by UIC health sciences faculty over a 15-year period and the results of survey of health science faculty use and perceptions of the University Library's services and resources.

### Research Setting

The University of Illinois Chicago is a large urban Research 1 university with one multidisciplinary library and one health sciences library found on its Chicago campus. Regional health sciences campuses are in Peoria and Rockford, and each include a UIC health sciences library providing access to the same online resources and services. Applied Health Sciences, Dentistry, Medicine, Nursing, Pharmacy, and Public Health are among the health sciences disciplines. While the University also considers Social Work a health sciences college, because this college is serviced by the multidisciplinary library, it was not included in this study.

The UIC library (all libraries) had approximately 15,948 active print collections in 1995. In 1998, the library subscribed to 15 online biomedical journals; by 2000, the library subscribed to more than 3,000 online journals. By 2008, the library had 25,000 online journals and by 2019, there were 28,000 online journals available through the University Library, increasing the availability of journal literature through the library's subscriptions. Database availability increased over time including free search tools (PubMed, Google Scholar), as did open access journals over time. The library had a subscription to Web of Science and Ovid MEDLINE prior to 2000, and in 2004, also added Scopus. While these variables are not controlled for in this study, their availability likely influenced faculty behavior.

## DATA COLLECTION

### Publication Data

To explore how publication patterns of faculty have changed over time and to explore the relationship between literature use and productivity, searches were conducted in 2020 in Scopus to capture bibliographic records for each faculty publication published between 2005 and 2019, including the number of references used in each publication. Each research team member was provided with a list of UIC faculty members who had been at UIC for at least 5 years, assigned to them for data collection. The team member utilized the “authors” search option in Scopus and typed in the faculty member's last name and initial name to retrieve the publication data from Scopus. Publication data was limited to the document type “article”, which filtered out non-journal publications (books and book chapters) and other publication types such as review articles, conference papers, letters, and editorials. The team member exported the list of publications including the citation information (authors, title, journal name, volume, issue, pages, DOI) and “funding details” into a spreadsheet after selecting all publications that met the requirements for the author). The team member then clicked on each article to find out how many references each publication's author(s) had used. More details about the data collection can be found in an Association of Research Libraries report summarizing the findings [12].

In addition to downloading the bibliographic records of each faculty members publications, the following information was captured: literature use (measured by number of references in the publications), grant funding (measured by whether the article was funded), and co-authorship size (measured by number of co-authors). A separate document summarized per faculty member, faculty productivity (measured by number of publications per faculty member), the average number of references included in each publication, and the average number coauthors. This document also included faculty demographics (e.g., status, college, and years at the institution), which were obtained from UIC's Office of Institutional Research (OIR).

### Survey Data

The University Library distributed a faculty survey in Spring 2022 using Qualtrics. There were 12 questions in the faculty survey, both open-ended and multiple choice. The survey focused on the use and importance of library resources and services as they relate to faculty teaching and research. The OIR was contacted to obtain UIC faculty demographic information and email addresses. In addition, faculty publication data (the number of articles, conference proceedings, books, and book chapters published in the last five years) for each faculty member was obtained from the University's faculty research management system. Participant demographic and publication data was uploaded into a panel in Qualtrics prior to survey distribution. Following UIC's Institutional Review Board (IRB) approval, the survey was sent to around 4,500 university faculty and postdoctoral employees at UIC between February 21 and March 25, 2022. As faculty respondents completed the survey, their de-identified demographic and publication data were added to their anonymous survey responses. More information on the development of the survey and a copy of the survey can be found in Scoulas & De Groote (2023) [13].

## RESULTS

### Publication Patterns

By exploring health sciences faculty that had been at UIC for at least 15 years, we were able to observe the publication patterns of the same faculty over a 15-year period. We calculated the average publications per faculty, the average number of references used in publications, and the average number of co-authors included in publications in 5-year intervals over a 15-year period by college.

In general, the number of publications per author, the number of references per publication and the number of co-authors per publication increased over time (Table 1). The perceptible exceptions to this general trend included the number of references decreasing in publications in nursing from 2010/2014 to 2015/2019 and a decrease in publications for pharmacy faculty, also from 2010/2014 to 2015/2019. Nursing had the most productive faculty as measured by publication output. Nursing, followed by pharmacy, used the most references in their publications. Medicine on average had the most co-authors.

**Table 1:** Publication patterns of tenure system health sciences faculty by college over 15 years.

	2005–2009	2010–2014	2015–2019	Avg all Years
	M (SD)	M (SD)	M (SD)	M
**Applied Health Sciences (n=11)**				
Publications per Faculty	14.64 (4.91)	18.46 (8.76)	19.73 (11.87)	17.61
References per Publication	39.75 (5.50)	39.56 (5.88)	44.49 (10.93)	41.27
Co-Authors per Publication	4.49 (1.12)	5.49 (1.26)	6.50 (3.61)	5.49
**Dentistry (n=15)**				
Publications per Faculty	7.87 (6.17)	11.27 (8.34)	10.13 (10.40)	9.76
References per Publication	36.13 (11.19)	37.69 (9.46)	45.13 (11.87)	39.65
Co-Authors per Publication	4.93 (1.39)	6.00 (1.24)	6.63 (2.00)	5.85
**Medicine (n=136)**				
Publications per Faculty	11.40 (8.33)	12.87 (9.49)	14.70 (14.27)	12.99
References per Publication	35.25 (14.49)	37.73 (14.46)	43.72 (16.96)	38.90
Co-Authors per Publication	7.25 (8.69)	7.32 (3.96)	10.74 (12.88)	8.44
**Nursing (n=6)**				
Publications per Faculty	9.83 (5.15)	18.17 (8.61)	24.83 (11.96)	17.61
References per Publication	47.07 (8.15)	51.83 (7.71)	47.09 (8.15)	48.66
Co-Authors per Publication	5.25 (1.87)	6.12 (1.52)	6.45 (0.76)	5.94
**Pharmacy (n=18)**				
Publications per Faculty	14.17 (11.02)	19.33 (12.80)	18.10 (12.64)	17.20
References per Publication	36.55 (7.36)	42.81 (9.17)	47.70 (11.49)	42.35
Co-Authors per Publication	5.54 (1.40)	7.03 (1.99)	18.56 (46.19)	6.75
**Public Health (n=24)**				
Publications per Faculty	10.42 (7.43)	11.79 (9.09)	13.25 (9.10)	11.81
References per Publication	35.19 (10.74)	37.25 (13.28	38.05 (10.37)	36.83
Co-Authors per Publication	5.08 (2.04)	5.78 (2.02)	6.05 (2.25)	5.64

The use of references included in the 2010 to 2019 publications was examined in correlation with the productivity of health sciences faculty, measured by the number of publications to explore the relationship between the use of literature and productivity. The results indicate that there were no statistically significant correlations between them (r [343] =0.09, p =.11). Despite this, it is important to know that the ratio of average references used appeared to be less for the least productivity faculty (avg. 37.2 references/article) compared to the more productivity (avg. 41.7 references/article).

As the publication patterns reported above were limited to journal publications, the broader spectrum of the publication output types were explored using the faculty publication data obtained from the faculty survey. Like the publication data presented above, the data was limited to tenure system health sciences faculty. While this data includes a greater number of faculty than the Scopus publication data and the timeline (2017 to 2022) is not an exact match to the data presented in Table 1 (2015 to 2019 data), it provides insight into faculty productivity beyond journal articles. The number of publications (books, book chapters, conference proceedings, and journal articles), was averaged by college. Table 2 shows that overall, faculty from Pharmacy published the most (M = 40), followed by those from Applied Health Sciences (M = 39), whereas faculty from Dentistry published the least (M = 19). Journal articles were the most common publication type for all health sciences colleges with faculty from Pharmacy and Applied Health Sciences publishing the most per faculty member, followed by nursing. Conference Proceedings are a more common publication output for Pharmacy faculty.

**Table 2:** Average health sciences tenure system faculty publication output type over 5-year period by college (2017 to 2021).

	N	Books	Book Chapter	Conference Proceedings	Journal article	Total all Publication
**Applied Health Sciences**	42	0.12	1.86	3.88	33.10	38.95
**Dentistry**	50	0.16	1.34	1.04	16.48	19.02
**Medicine**	605	0.07	1.78	3.20	19.08	23.53
**Nursing**	34	0.00	0.68	3.44	25.71	29.82
**Pharmacy**	46	0.13	1.70	7.26	30.37	39.46
**Public Health**	48	0.08	0.52	2.71	18.63	21.94

**Table 3:** Correlations between faculty's perceptions on the importance of and use of the library resources and services and their level of research productivity.

	Print books	eBooks	Journals	Databases	Special Collections	Inter-library Loan	Librarian Assistance	Literature Search Support
**Perception of the importance of the library resources and faculty research productivity**								
	(n=193)	(n=202)	(n=211)	(n=209)	(n=187)	(n=203)	(n=197)	(n=204)
**Publications (2021)**	0.092	0.117	0.091	−0.044	−0.005	0.089	0.025	−0.036
**Publications (2017–2021)**	0.098	−0.121	0.079	−0.083	−0.061	0.024	−0.015	−0.066
**Use of library resources and services and faculty research productivity**								
	(n=194)	(n=199)	(n=204)	(n=204)	(n=195)	(n=201)	(n=199)	(n=202)
**Publications (2021)**	0.026	−0.127	.145*	−0.025	0.010	.149*	0.049	0.057
**Publications (2017–2021)**	−0.040	−0.139	.141*	−0.056	−0.023	.165*	0.003	0.004

p < .05 level. Research productivity includes books, book chapters, conference proceedings, and journal articles.

### Survey Responses

A total of 557 university faculty members out of 4,507 responded to the survey (12.4% response rate). Of those, 267 health science faculty out of 2,689 (9.9%) responded to the survey. Forty percent of health sciences faculty respondents were assistant professors, followed by professors (23.6%), associate professors (21.4%), and instructors and lecturers (15%). More than half of the faculty were from Medicine (54%), followed by Pharmacy (13%), Applied Health Sciences (10.5%), Nursing (9.4%), Public Health (7.1%) and Dentistry (5.6%). On average, health sciences faculty had worked at the institution for about 11 years.

**Figure 1:**
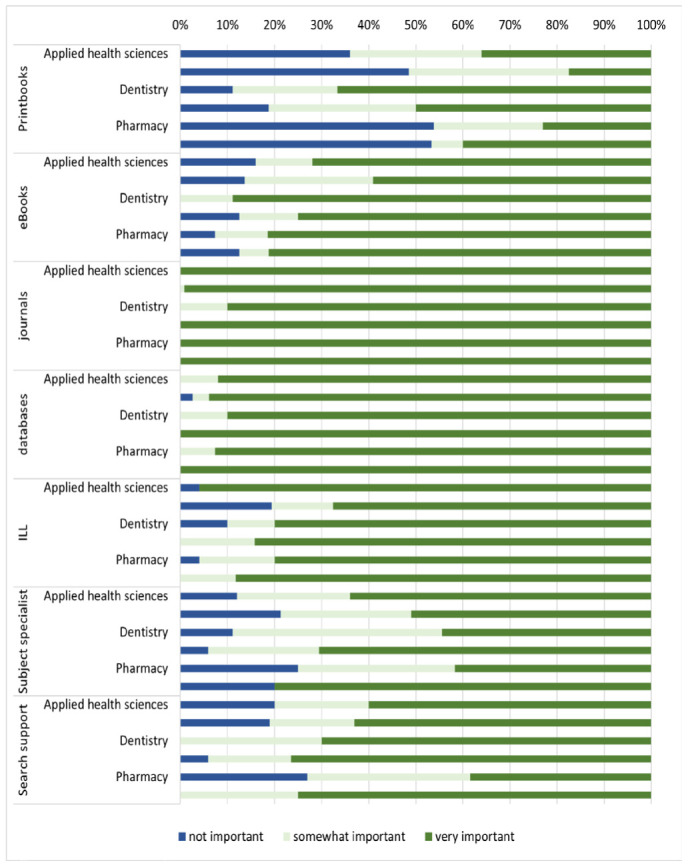
Importance of library resources to research by college.

Faculty members rated the importance of seven listed library services and resources on a scale of 1 (not at all) to 9 (extremely), which were then grouped into three categories: 1-3 for not important, 4-6 for somewhat important, and 7-9 for very important (Figure 1). The results indicate that most faculty members rated online journals and databases as the most important resources, followed by interlibrary loan (ILL). When looking at the resources by college level, almost all faculty perceived the journal as very important regardless of the discipline. However, perceptions of faculty members regarding databases and ILL differed slightly by college level. Faculty members from nursing and public health rated databases as the most important, whereas those from medicine rated them as the least important. For ILL, faculty members from applied health sciences perceived it as the most important, while those from medicine rated it as the least important.

**Figure 2:**
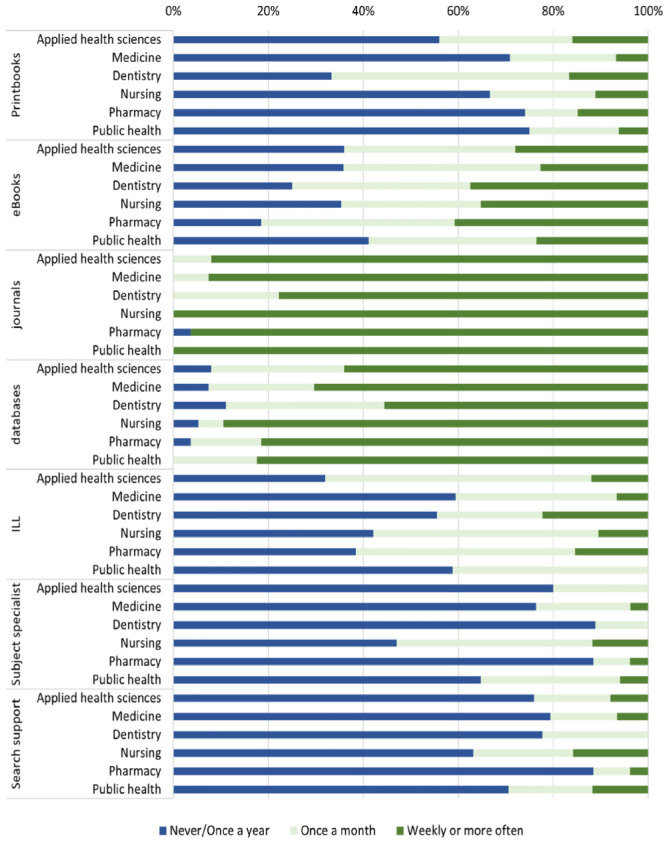
Frequency of library resource use for research purposes by college.

Faculty members were asked to rate how often they used seven resources and services provided by the library (Figure 2). The study found that journals and databases were the most frequently used resources, followed by eBooks. Their frequency of use varied by college level, which is consistent with the faculty members' perceptions of the importance of those resources. Faculty members from nursing and public health were found to use journals and databases the most frequently, while those from pharmacy used journal the least, and those from dentistry used database the least. For eBooks, faculty from pharmacy used it the most, followed by those from dentistry. For ILL, despite the faculty members' perceptions of its importance, their actual use of this resource differed. Faculty members from applied health science were recorded as the most frequent users (68% for monthly or more), whereas those from medicine were the least frequent users (41% for monthly or more).

### Faculty Productivity and Importance and Use of Library Resources and Services

The faculty members were asked to assign a rating, ranging from 1 to 9, to reflect their views on the significance of library resources, with 9 representing an extremely important rating, and 1 indicating a not at all important rating. The study then explored whether there was a relationship between the faculty members' views on the value of library resources for research and their level of research productivity, which was measured by the number of publications (such as journal articles, conference proceedings, books, and book chapters) between 2017 and 2021. The results of the Pearson correlation analysis revealed no significant correlation between the faculty members' perceptions of library resources and their research productivity.

To investigate whether there was a relationship between faculty members' frequency of library resource use and their research productivity, the study conducted a Spearman rank correlation analysis. The research productivity was measured by the number of publications (including books, book chapters, conference proceedings, and journals) in 2021 and the 5-year period from 2017 to 2021. The findings suggest that certain library resource uses, specifically online journals (rs [204] = .145, p < .05 for 2021 and rs [204] = .141, p < .05 for the 5-year period) and interlibrary loan (ILL) (rs [201] = .149, p < .05 for 2021 and rs [201] = .165, p < .05 for the 5-year period), were correlated with research productivity. This implies that higher usage of online journals and ILL was linked to a greater number of publications in 2021 and during the 5-year period from 2017 to 2021.

## DISCUSSION

Health sciences faculty publication patterns have changed over time, marked by an increase in productivity, collaboration, and literature used in publications. Undoubtedly, the increase and availability of online journals, open access journals, and online databases has facilitated the access to and use of online journals, thus increasing the literature used in publications over time.

Other factors not explored in this analysis may also have had an impact. For example, grant funded publications and publications with more co-authors also tend to include more references than non-grant funded publication [12]. Faculty productivity also varies by college, although different levels of productivity were observed between the two datasets exploring productivity. The data set looking at faculty over time (15-year period) would have focused largely on mature researchers whereas the largest faculty group participating in the survey was assistant professors, which may have influenced the productivity observed. However, the broader publication data demonstrated that health sciences faculty also have their scholarship published in conference proceedings, books, and book chapters, although these publication output types are limited and vary by college.

While differences existed in how the health sciences colleges perceived the importance of various library resources, all colleges reported that online journals and databases were very important to their research. They were often used by faculty in most colleges at least monthly. ILL was also considered an important resource by faculty in most colleges, although use of the service varied by college. In general, the importance of library resources was similarly reflected in their use. While faculty in most colleges rated services provided by library professionals (subject specialist and search support) as somewhat or very important, they were general less likely to use these services, suggesting that librarians' expertise is valued, but faculty are generally independent searchers. Faculty may like to know that they can call upon help when needed. Although the scales and some of the resources asked about were different from the current study, Inman et al (2019), also found that faculty considered journals and internet resources as important for conducting research and library databases such as PubMed and interlibrary loan were very important in meeting faculty information needs [11]. In a similar vein, faculty in both studies did not rank books as important as other information resources, although eBooks were rated in both cases as more important than print books. High use of ILL by applied health sciences faculty suggests that the library's journal collection may not being meeting their needs, while faculty in medicine may be finding that most of their needs are met with the library's collection given their low ILL use.

Both data sets suggest that faculty's use of the literature appears to have a relationship with productivity. Survey results demonstrated a relationship between productivity and use of online journals and ILL, where those with more publications reported greater use of online journals and ILL. Although there were no statistically significant correlations between the use of literature and faculty's research productivity, the descriptive statistics from the publication data indicate that those faculty who were less productive also used less references in their publications.

Faculty who were productive and prolific used more references, although the prolific authors tended to use less references than the productive authors. More exploration is needed to understand why highly productive faculty use less literature in their publications. Similarly, further exploration is needed, as it relates to the decreased literature use in publications between 2015 and 2019 for faculty from nursing.

## LIMITATIONS

In both data sets, there are some limitations. The survey data only represents faculty that agreed to participate in the survey. Therefore, observed results may not necessarily reflect the characteristics and behaviors of the broader body of health sciences faculty at the institution in the study. For the publication data set that focused on health sciences faculty at the institution for 15 years, these faculty would have also matured in their research over time, which may have some impact on the relative increase in publications over time. In addition, because faculty needed to be at UIC for at least, it limited the number of faculty that could be included, which impacts the generalizability of the data in some colleges. For example, the college of Nursing had a small sample of only 6 researchers.

## CONCLUSIONS

The results from the publication and survey provide some insights into health sciences faculty information-seeking behavior and productivity. Libraries' efforts to provide seamless access to information have been successful, as illustrated by the increased use of references in publications. While many factors such as increased online and freely available resources also likely play a role in this, the library remains a critical and valued resource for faculty research. The results also show that library resources such as online journals and databases are still very important to faculty research. Use of the literature appears related to productivity, where productive faculty are more likely to use online journals.

While resources such as print and electronic books are less important than journals and databases for conducting research, they are still utilized by all disciplines. In addition, faculty value the expertise provided by library professionals even if they were not widely utilized. In times of limited budgets and the perception that information is freely available to all, documenting and demonstrating the valuable role library resources play in supporting faculty research is critical.

The results of this study also confirm that publication patterns change over time, demonstrating an increase in publications overall and an increase in co-authorship on those publications. Productivity, literature use, and co-authorship varied by college. Academic libraries can play a crucial role in supporting their university's research planning and endeavors by exploring and demonstrating changing scholarly publication patterns and engagement. Furthermore, they can assist in understanding their faculty's publication patterns, providing valuable insights that can be used to shape future research initiatives and enhance the overall quality of scholarship.

## DATA AVAILABILITY STATEMENT

Data associated with this article are available in the authors' institutional repository: ARL study data: https://doi.org/10.25417/uic.24891984.v1; 2022 Faculty survey and data: https://doi.org/10.25417/uic.23549247.

## AUTHOR CONTRIBUTIONS

Sandra De Groote: conceptualization; data curation, formal analysis; investigation, methodology; project administration, visualization; writing - original draft. Jung Mi Scoulas: conceptualization; data curation, formal analysis; investigation, methodology; project administration, visualization; writing - review & editing.

## References

[1] De Groote SL, Dorsch JL. Online journals: impact on print journal usage. Bull Med Libr Assoc. 2001 Oct;89(4):372. 11837259 PMC57966

[2] Tenopir C, King DW, Spencer J, Wu L. Variations in article seeking and reading patterns of academics: What makes a difference?. Libr Inf Sci Res. 2009 Sep;31(3):139–48.

[3] Tenopir C, Mays R, Wu L. Journal article growth and reading patterns. New Rev Inf Netw. 2011 May;16(1):4–22.

[4] Tenopir C, Christian L, Kaufman J. Seeking, reading, and use of scholarly articles: An international study of perceptions and behavior of researchers. Publications. 2019 Mar;7(1):18.

[5] Haglund L, Olsson P. The impact on university libraries of changes in information behavior among academic researchers: a multiple case study. J Acad Libr. 2008 Jan;34(1):52–9.

[6] Ollé C, Borrego Á. A qualitative study of the impact of electronic journals on scholarly information behavior. Libr Inf Sci Res. 2010 Jul;32(3):221–8.

[7] De Groote SL, Shultz M, Blecic DD. Information-seeking behavior and the use of online resources: a snapshot of current health sciences faculty. J Med Libr Assoc. 2014 Jul;102(3):169. 25031557 10.3163/1536-5050.102.3.006 PMC4076125

[8] Blankstein, M, Wolff-Eisenberg C. Ithaka S+ R US library survey 2018. Ithaka 2019 10.18665/sr.311199.

[9] De Groote SL, Barrett FA. Impact of online journals on citation patterns of dentistry, nursing, and pharmacy faculty. J Med Libr Assoc. 2010 Oct;98(4):305. 20936070 10.3163/1536-5050.98.4.008 PMC2947137

[10] De Groote SL. Citation patterns of online and print journals in the digital age. J Med Libr Assoc. 2008 Oct;96(4):362. 18974814 10.3163/1536-5050.96.4.012 PMC2568853

[11] Inman M, Blevins AE, Ketterman E, Young KL. Now tell us what you want: information-seeking habits of health sciences faculty. Med Ref Serv Q. 2019 Apr;38(2):131–42. 31173574 10.1080/02763869.2019.1588046

[12] De Groote S, Scoulas JM, Dempsey P, Blecic DD, Barrett F. Library Impact Research Report: Faculty Publication Patterns at a Large Urban University and Correlation with Collections Use and Size. Washington, DC: Association of Research Libraries, 2022. 10.29242/report.uillinoischicago2022.

[13] Scoulas JM, De Groote SL. Faculty perceptions, use, and needs of library resources and services in a public research university. J Acad Libr. 2023 Jan;49(1):102630.

